# Health diagnosis of coastal zone ecosystem: China's case

**DOI:** 10.3389/fpubh.2023.1038761

**Published:** 2023-02-02

**Authors:** Cai Zhang, Miao Wang

**Affiliations:** ^1^Business College, Qingdao University, Qingdao, Shandong, China; ^2^Management College, Ocean University of China, Qingdao, Shandong, China

**Keywords:** coastal zone ecosystem, ecosystem health model, state space model, ecological health early warning, Shandong Province

## Abstract

With its unique geographical location, the coastal zone has concentrated great advantages in resources, population and economy. However, with the deepening of marine resources development activities, a series of problems have emerged in the coastal zone, such as the gradual shortage of resources, the decline of environmental quality and the increase of ecological risks. The coastal zone ecosystem has shown a certain degradation trend. Maintaining the health of the coastal zone ecosystem has become the primary task of the sustainable development of the marine economy. Monitoring the coastal ecosystem carrying capacity, diagnosing the health status of the coastal ecosystem, effectively planning and managing the development and utilization of natural resources in the coastal zone, and controlling human activities related to the sea within the ecological carrying capacity and health limits of the coastal zone are of great significance to the sustainable development of society and economy in the coastal zone. In this study, the ecosystem health model, state space model and ecological health early warning principle were comprehensively applied to construct the coastal ecosystem health diagnosis framework of “Carrying type **→**Early warning degree **→**Health level;” The evaluation index system of coastal ecosystem carrying capacity was established; Taking Shandong Province as the empirical research object, the health status of the coastal ecosystem in Shandong Province was diagnosed by using the relevant data from 2007 to 2019. The empirical results show that: (1) From 2007 to 2013, the carrying capacity of the coastal ecosystem in Shandong Province was relatively good, in the state of “loadable” or “critical overload,” while from 2014 to 2019, the carrying capacity was poor, in the state of “overload;” (2) From 2007 to 2013, the early warning degree of coastal ecosystem health in Shandong Province was in the state of “no alarm,” “light alarm,” and “medium alarm,” and the health level was in the state of “very healthy,” “healthy,” and “sub-health;” from 2014 to 2019, the health warning level of the coastal ecosystem in Shandong Province was in the state of “serious alarm” and “extremely serious alarm,” and the health level was in the state of “unhealthy” and “Morbid,” and the health status was worrying; (3) The key influencing factors affecting the carrying capacity of the coastal ecosystem mainly included the output of marine mining, marine GDP, per capita marine production, total amount of main pollutants directly discharged into the sea, domestic tourist arrivals in coastal cities, area of marine nature reserves, proportion of class IV and inferior class IV seawater, average density of beach garbage in the monitoring area, number of medical and health institutions; Finally, some policy suggestions were put forward to improve the health of coastal ecosystem in Shandong Province. In the “discussion” part of this study, the consistency between the research results of this paper and the actual situation of the marine ecosystem in Shandong Province and the existing research results of the same kind is compared, and the applicability and limitations of the research methods in this paper are put forward, indicating that the research methods in this paper are more applicable to the comparative analysis under the same ideal value determination criteria.

## 1. Introduction

The coastal zone is the transition zone of the interaction between the sea and the land, the ecological fragile zone with intensive human activities, and the most active area of social economy. More than 50% of the world's population lives in coastal areas, which account for 20% of the earth's surface area. With the continuous expansion of human activities to the ocean, about 41% of the global coastal zone is strongly affected by human activities (1). About 60% of the population and two-thirds of the large and medium-sized cities are gathered in the coastal areas in China. In recent years, the urbanization and industrialization of the coastal zone have been accelerated. However, the lack of scientific planning for regional development has led to excessive spatial competition, high pressure on resources and environment, unreasonable regional industrial structure, and continuous increase in the total amount of pollution emissions, which have an increasingly serious impact on the coastal ecosystem (2). As the first marine economic zone, the coastal zone is not only the precious land resources of coastal countries, but also the base of marine development and economic development, it plays a very important role (3).

The coastal ecosystem has the characteristics of complex, marginal and active. At the same time, the coastal ecosystem is affected by the land and marine environment. It is sensitive to environmental disturbance and has poor system stability. It is a typical fragile ecological environment area. The coastal zone is also the zone with the most abundant resources, the most obvious location advantages, the most fragile ecology and more disasters. Due to the strong interaction between land and sea, and the interaction of natural factors and human factors, the ecological environment in the coastal zone is inherently fragile. If it is not developed and utilized properly, it will bring more complicated negative environmental effects and it will be very difficult to repair. The destruction and loss of the coastal ecological environment will inevitably lead to increased pollution, regional environmental deterioration, and serious threats to biodiversity and aquatic resources. The coastal ecosystem health diagnosis can not only guide the decision-making of coastal regional development planning, but also provide necessary decision-making support for the protection and restoration of coastal ecosystem (4).

There are many fields of ecosystem health research. For example, on the research of the regional ecosystem health, Ayinuer et al. used the integrated ecosystem vitality-ecosystem organic-ecosystem persistence (V-O-E) model to assess the ecosystem health in Central Asia (5); Shi et al. evaluated the ecosystem health of pastoral areas on the Qinghai Tibet Plateau based on the multi model combination method (6). In the rural ecosystem health, Liu et al. used the REH-SD assessment framework to build an ecosystem health assessment index system for rural areas in mountainous areas of Chongqing (7); Tu et al. constructed the indicator system and indicator selection model of rural ecosystem health assessment in Luoshan County (8). In the urban ecosystem health, Wang et al. evaluated the ecosystem health of Jining City by combining energy analysis indicators with traditional economic and social indicators (9); Shen et al. used the improved SI-MI model to evaluate the health status of the urban agglomeration ecosystem in the middle reaches of the Yangtze River (10). In the land ecosystem health, Guo et al. built an indicator system for land ecosystem health evaluation based on the improved PSR model, and evaluated the land ecosystem health in Chang Zhu Tan region (11); Yang et al. used the evaluation framework of pressure, state (vitality- organization-resilience-function) and response to evaluate the health level of Qiqihar's land ecosystem (12). In the forest ecosystem health, Chen et al. constructed the remote sensing diagnostic index system of forest ecosystem health in the forest region by using the analytic hierarchy process, and diagnosed the health status of the forest ecosystem in Baili Azalea Forest region (13); Zhou et al. used VOR comprehensive index model to evaluate the forest land health in Altai Mountain (14). In the grassland ecosystem health, Zhang KL, et al. used VOR model to evaluate the grassland ecosystem health in Qinghe forest region of Altai Mountain in Xinjiang (15); Chen et al. used the chromatography analysis method and VOR comprehensive index model to evaluate the grassland ecosystem health in Xinjiang (16). In the wetland ecosystem health, Zhang SY, et al. constructed a comprehensive assessment model of wetland ecological health based on the basic principle of Weber Fechner Law to assess the wetland ecological health in Qinmang River basin (17); Zhang MY, et al. used the comprehensive health index to evaluate the ecosystem health level of Yuehai National Wetland Park (18). Therefore, ecosystem health research covers many fields.

On the research of marine ecosystem health, Wu et al. used the framework of marine health index (OHI) to assess the health status of Shanghai coastal ecosystem (19); Rombouts et al. used direct observation and modeling methods to evaluate the health of marine ecosystems (20); Zheng et al. used the marine health index to quantitatively assess the health of Tianjin's marine ecosystem (21); Joesidawati et al. used the IKLI (Indeks Kesehatan Laut Indonesia) framework to assess the health of the coastal ecosystem in Tuban, East Java Island (22); Li et al. used the gray system method to evaluate the health of offshore marine ecosystems (23). In the bay ecosystem health, Song et al. used the Analytic Hierarchy Process (AHP) to evaluate the health of the Laizhou Bay ecosystem (24); Seung et al. assessed the ecosystem health status of Guangyang Bay and Golden Bay in South Korea based on the biological integrity planktonic index (P-IBI) (25). In the coral ecosystem health, Brenda. et al. used the nutrient model to assess the system structure, organization and health status of coral ecosystems in three marine protected areas (MPA) along the tropical Pacific coast of Mexico (26); Ji et al. used the pressure state response (PSR) model to build an evaluation index system to evaluate the health of typical coral reef ecosystems in China (27). In the ecosystem health of the marine park, Johan Danu Prasetya et al. used the analytic hierarchy process (AHP) to evaluate the coastal ecosystem health of Karimonjawa National Marine Park (28); Prasetya et al. used the mangrove health index to evaluate the health of mangrove ecosystem in Karimonjawa National Marine Park, Indonesia (29). In the island ecosystem health, Joesidawati et al. used the marine health index to measure the health status of the coastal ecosystem in Tuban, East Java Island, Indonesia (22); Zhang H, et al. used principal component analysis (PCA) to build an island ecosystem assessment framework to assess the ecosystem health of Zhoushan Islands (30). In the ecosystem health in special marine regions, Wang et al. built an indicator system for ecosystem health assessment of marine ranching based on PSR model (31); Ding et al. used cluster analysis and Marine Biotic Index (M-AMBI) and other methods to assess the health status of the benthic ecosystem in Jiaozhou Bay (32); Zhao et al. used RS and GIS technology and PSR conceptual model to evaluate the ecosystem health of Ningde Marine Ecological Special Reserve in Fujian Province (33). It can be seen that the research on the health of marine ecosystem is also multifaceted.

The above research results have laid a good foundation for the research on the health of various ecosystems, but most of them are based on the application or improvement of a method, with few comprehensive studies. In this study, the ecosystem health model, the state space model and the ecological health early warning principle were comprehensively used to build a chain type coastal zone ecosystem health diagnosis framework of “carrying capacity → early warning degree → health level,” and an evaluation index system of coastal zone ecosystem carrying capacity was established. The purpose of this study is to explore the methods of coastal ecosystem health diagnosis from a new perspective, emphasizing a step-by-step and hierarchical diagnosis process, so as to provide more scientific and reasonable decision support. At the same time, it also provides suitable reference for health diagnosis of other ecosystems.

## 2. Research methods

### 2.1. Ecosystem health model

**Figure 1:**
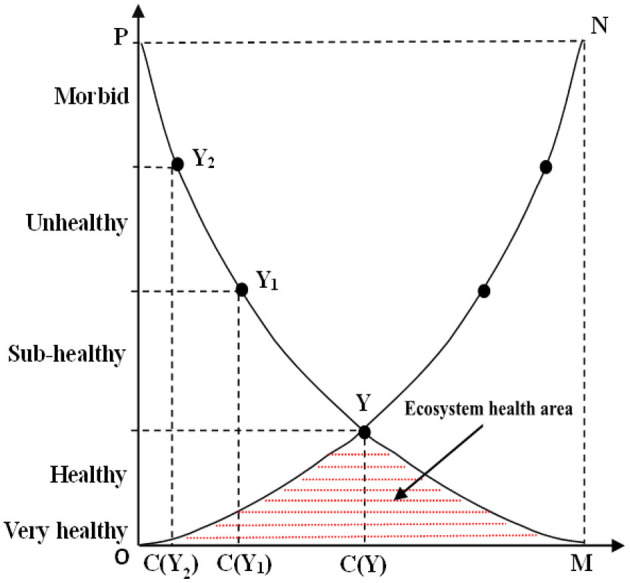
Ecosystem health model.

Ecological carrying capacity is an important standard to measure ecosystem health. Figure 1 describes the relationship between ecosystem health and ecological carrying capacity (34–37). For any relatively independent ecosystem, a certain level of ecological carrying capacity and the level of social and economic pressure bear correspond to a certain level of ecosystem health.

In Figure 1, ON is the carrying capacity curve and PM is the pressure curve. C (Y), C (Y1), and C (Y2) respectively represent the standard values of ecological carrying capacity in healthy, sub-healthy and unhealthy states. OYM is an ecosystem health area, indicating that the health status corresponding to human activity pressure and ecological carrying capacity is at a healthy level; OPY is a restricted area of “lack of carrying capacity,” and the social and economic pressure can only be strictly controlled below the OY line to ensure the health of the whole ecosystem; NYM is the restricted area of “excessive pressure,” and the reduction of carrying capacity shall be controlled below the YM line. Under the two conditions of “lack of carrying capacity” and “excessive pressure,” if the critical line is broken, the health level of the ecosystem will be reduced, and in serious cases, the ecosystem may be degraded. The pressure of excessive human activities in the system will cause the health critical Y-point to move down, the ecological health area to shrink, and the health level corresponding to the original carrying capacity level to decline. On the contrary, the improvement effect of human activities on the ecosystem will move the Y-point upward and raise the health level corresponding to the original carrying capacity level. Therefore, the health status corresponding to stress and ecological carrying capacity is in a healthy or higher level, which is the necessary condition for ecosystem health.

### 2.2. State space model

Ecological carrying capacity is an important evaluation index to measure the sustainable development of ecosystem. Therefore, scientific assessment of the ecological carrying capacity of the coastal zone and control of human activities within the anti-interference regulation capacity of the coastal zone ecosystem have important practical significance for the sustainable development of the coastal zone composite ecosystem (38).

In this study, the state space model is used to study the carrying capacity of coastal ecosystem. State space method is a kind of European geometric space method, which can quantitatively describe the state of a system. Ecological carrying capacity based on ecosystem health is to explore the potential ability of natural ecosystem to maintain its service function and its own health under certain social and economic conditions by taking ecosystem as the research object (39).

**Figure 2:**
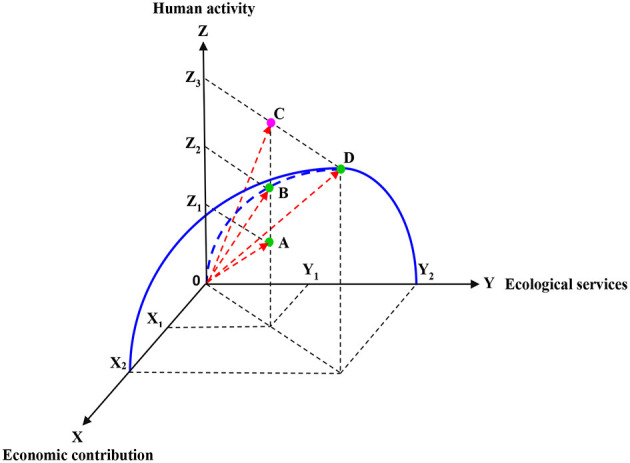
State space model.

The development and utilization of coastal zone resources can bring economic benefits to mankind. The greater the development intensity, the greater the economic benefits and the greater the impact on the ecological environment. Therefore, the economic contribution of coastal zone to human is restricted by the carrying capacity of coastal zone ecosystem. The ecological service function of the coastal zone should be brought into play within the carrying capacity of the coastal zone ecosystem, otherwise the health of the coastal zone ecosystem will be affected. Human activities of exploiting and utilizing coastal resources are the direct cause of the degradation of coastal ecological environment. Therefore, in this study, a spatial state model composed of three subsystems of coastal zone economic contribution, ecological service and human activities constructed, as shown in Figure 2.

In the state space model, the ecological carrying capacity of the coastal zone is represented by the vector module in the state space, that is, the vector is formed by the state point in the space and the origin of the system. In the state space, any carrying state of the coastal ecosystem in a certain space-time scale can be represented by a point in the state space. The vector modules (such as OA, OB, and OC) formed by the origin O and the state point respectively represent the values of the ecological carrying capacity of the coastal zone.

X_2_OY_2_ constitutes the curved surface of ecological carrying capacity of the coastal zone, and the points A, B, C and D represent different carrying conditions, respectively. Any point lower than the curved surface indicates that human activities related to the sea have not reached the ecological carrying capacity limit of the coastal zone under a specific ecological environment combination (such as point A); Any point higher than the curved surface indicates that human activities related to the sea have exceeded the carrying capacity limit of the coastal zone under a specific ecological environment combination (such as point C); Any point on the curved surface (such as point B) indicates that the impact of human activities on the coastal ecosystem has reached the limit of ecological carrying capacity, among them, the vector value of AD is the largest, and point D is the largest carrying state point on the curved surface. In Figure 2, three status points A, B and D are “non-overload points” while the status point C belongs to “overload points.” The points in the state space represent the combination of economic contribution, ecological services and human activities in the coastal zone, and the carrying states of these points at their locations are shown in Table 1.

**Table 1:** Carrying states of different points in the state space model.

**Point**	**Position**	**Carrying condition**	**Carrying status**
A	Below the carrying surface	The impact of human activities is lower than the ecological carrying limit of the coastal zone	Loadable
B	On carrying surface	The impact of human activities reaches the ecological carrying limit of the coastal zone	Fully loaded
C	Above the carrying surface	The impact of human activities exceeds the ecological carrying capacity of the coastal zone	Overload

Therefore, the ecological carrying capacity of the coastal zone can be expressed by using the vector modulus formed by the origin in the state space and the system state point, the mathematical expression is:


(1) $$\begin{array}{l} ECC=\;M=\sqrt{\overset{n}{\underset{i=1}{\sum }}{\left(w_{i}E_{i}\right)}^{2}+\overset{m}{\underset{j=1}{\sum }}{\left(w_{j}R_{j}\right)}^{2}+\overset{p}{\underset{k=1}{\sum }}{\left(w_{k}H_{k}\right)}^{2}\;} \end{array}$$

Where, ECC (Ecological Carrying Capacity) is the value of ecological carrying capacity of coastal zone; M is the modulus of the spatial vector of the ecological carrying capacity of the coastal zone; *E*_*i*_ is the projection of the *i*th coastal zone economic contribution index on the spatial coordinate axis; *R*_*j*_ is the projection of the *j*th coastal zone ecological service index on the spatial coordinate axis; *H*_*k*_ is the projection of the *k*th human sea related activity index on the spatial coordinate axis; *w*_*i*_, *w*_*j*_, and *w*_*k*_ are the weights corresponding to the *i*th, *j*th, and *k*th indexes respectively; *n, m*, and *p* are the index numbers corresponding to the three subsystems of economic contribution, ecological services and human activities in the coastal zone.

The function expression is:


(2) $$\begin{array}{l} E=f\left(E_{1},E_{2},\ldots ,E_{n}\right) \end{array}$$

(3) $$\begin{array}{l} R=f\left(R_{1},R_{2},\ldots ,R_{m}\right) \end{array}$$

(4) $$\begin{array}{l} H=f\left(H_{1},H_{2},\ldots ,H_{p}\right) \end{array}$$

According to the principle of state space model, the actual value of coastal ecosystem carrying capacity is compared with the ideal value to determine the ecological carrying capacity of coastal zone. The ideal state refers to the state in which the interaction and coupling of “people” and “sea” in the coastal zone ecosystem reach the optimal combination within a certain period of time. This state indicates the amount of human social and economic activities that can be supported by the coastal zone resource environment under the premise of the existing economic and technological conditions and the level of human understanding, taking sustainable development as the criterion (40). Therefore, the modulus of the ecological carrying capacity vector of the coastal zone in “overload” state is necessarily greater than that of the ideal coastal zone carrying capacity vector; on the contrary, the modulus of the coastal ecological carrying capacity vector in “loadable” state is necessarily smaller than that in ideal state. According to the comparison between the modulus of the coastal zone ecological carrying capacity vector and the modulus of the ideal coastal zone carrying capacity vector, the carrying capacity of the coastal zone ecosystem can be judged.

Considering the complexity and dynamics of the coastal ecosystem, the setting of the carrying standard needs to have a certain tolerance. The ideal state of the coastal ecosystem carrying capacity is represented by a certain interval, which is an interval with a length of ±0.2 centered on the value of the coastal ecosystem carrying capacity in the ideal state. The judgment of the coastal ecosystem carrying status is mainly based on the comparison between the actual state value of the system carrying capacity and the ideal state carrying interval (41). The choice of ideal value depends on the specific situation, for example, if the average level of the indicators in the study period is taken as the evaluation standard, the average value of the indicators can be taken as the ideal value.

ECC^*^ represents the value of the coastal ecosystem carrying capacity vector in the ideal state, the value of the coastal ecosystem carrying capacity and carrying status can be obtained (42), as shown in Table 2.

**Table 2:** Value of vector modulus of coastal ecosystem carrying capacity and carrying status.

**Situation**	**Value of vector modulus**	**Carrying states**
1	0 ≤ ∣ECC∣ ≤ 0.8•∣ECC^*^∣	Light load
2	0.8•∣ECC^*^∣ < ∣ECC∣ ≤ 1•∣ECC^*^∣	Loadable
3	1•∣ECC^*^∣ < ∣ECC∣ ≤ 1.2•∣ECC^*^∣	Critical overload
4	1.2•∣ECC^*^∣ < ∣ECC∣ ≤ 1.4•∣ECC^*^∣	Overload
5	1.4•∣ECC^*^∣ < ∣ECC∣	Severe overload

^*^According to the principle of state space model.

### 2.3. Principle of ecosystem health early warning

**Figure 3:**
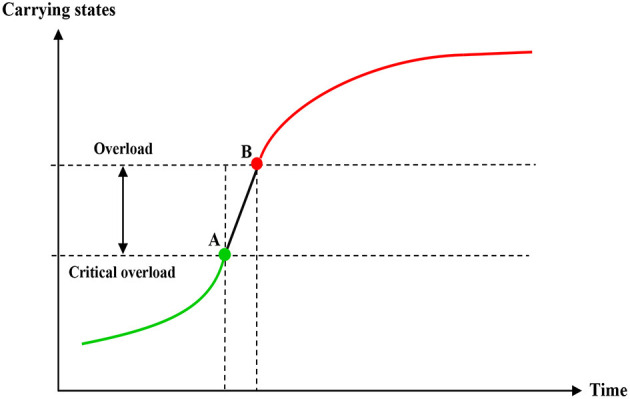
Principle of ecosystem health early warning.

On the basis of self-regulation, the capacity of the coastal ecosystem to bear the demand of human resources utilization and social and economic development is limited, while the human demand is unlimited. With the increase of ecological pressure, the ecological function of the ocean itself is continuously reduced and the environmental capacity is continuously reduced. In this process, two threshold critical point will appear, namely, critical overload point A and overload point B (see Figure 3), so as to give early warning of the actual carrying status of the coastal ecosystem, and then put forward targeted countermeasures and suggestions to provide basis for monitoring, feedback and regulation of the coastal ecosystem.

In September 2017, the Chinese government issued “several opinions on establishing a long-term monitoring and early warning mechanism for the carrying capacity of resources and the environment,” proposing that the carrying capacity of resources and the environment should be divided into three levels of “overload,” “critical overload,” and “non-overload.” According to the degree of aggravation and slowing down of the consumption of resources and the environment, the “aggravated overload” and “slowing overload” were divided into “red” and “orange” early warning levels, the “aggravated critical overload” and “slowing critical overload” were divided into “yellow” and “blue” early warning levels. The “no overload level” was determined as “green –no alarm level.” Therefore, the ecological early warning levels were “red,” “orange,” “yellow,” “blue,” and “green” from high to low, indicating different levels of alarm.

By comparing the carrying capacity of the coastal ecosystem in the previous year, the overload situation of this year is further judged, whether it is “aggravation” or “slowed down,” and defines five types of carrying capacity, early warning level and early warning degree, as shown in Table 3.

**Table 3:** Coastal ecological health early warning classification.

**Carrying type**	**Overload classification**			**Early warning level**	**Early warning degree**
No overload				Green warning	No warning
Critical overload	Current year value ≤ Last year value	↘	Critical overload (↘) (slowing critical overload)	Blue warning	Light warning
	Current year value ≥Last year value	↗	Critical overload (↗) (aggravated critical overload)	Yellow warning	Medium warning
Overload	Current year value ≤ Last year value	↘	Overload (↘) (slowing overload)	Orange warning	Heavy warning
	Current year value ≥Last year value	↗	Overload (↗) (aggravated overload)	Red warning	Extremely heavy warning

The green arrow represents “slow” and the red arrow represents “accelerated”.

According to the level of ecosystem health, the assessment standard of coastal ecosystem health can be divided into five levels of “very healthy,” “healthy,” “sub-healthy,” “unhealthy,” and “morbid” (34). See Table 4 for the meanings of each coastal zone ecosystem health level.

**Table 4:** Meaning of coastal ecosystem health level.

**Early warning degree**	**Health level**	**Meaning of health level**
No warning	Very healthy	The economic contribution is large; the ecological service function is strong; the impact of human activities on the ecological environment is small
Light warning	Health	The economic contribution is relatively large; the ecological service function is relatively strong; the impact of human activities on the ecological environment is relatively small
Medium warning	Sub-health	The economic contribution is general; the ecological service function is general; the human activities have a certain impact on the ecological environment
Heavy warning	Unhealthy	The economic contribution is relatively small; the ecological service function is relatively poor; the human activities have a relatively large impact on the ecological environment
Extremely heavy warning	Morbid	The economic contribution is very small; the ecological service function is very poor; the human activities have a great impact on the ecological environment

### 2.4. Index contribution rate

In order to reflect the impact of various indicators on the carrying capacity of coastal ecosystem, in this study, the contribution rate theory was introduced, and the contribution rate of the indicator carrying capacity is expressed by the percentage of the indicator carrying capacity in the subsystem carrying capacity (43). The calculation formula of the contribution rate is as follows:


(5) $$\begin{array}{l} \mu_{ECi}=\frac{EC_{i}}{ECC_{ec}}\times 100\% \end{array}$$

(6) $$\begin{array}{l} \mu_{ESj}=\frac{ES_{j}}{ECC_{es}}\times 100\% \end{array}$$

(7) $$\begin{array}{l} \mu_{HAk}=\frac{HA_{k}}{ECC_{ha}}\times 100\% \end{array}$$

Where, μ_*EVi*_, μ_*ESj*_, and μ_*HAk*_ respectively represent the index contribution rates of the three subsystems of coastal economic contribution, ecological services and human activities; *EC*_*i*_, *ES*_*j*_, and *HA*_*k*_ respectively represent the indicator carrying capacity of the three subsystems of coastal economic contribution, ecological services and human activities; *ECC*_*ec*_, *ECC*_*es*_, and *ECC*_*ha*_ respectively represent the carrying capacity of the three subsystems of economic contribution, ecological services and human activities in the coastal zone.

### 2.5. Index data standardization and weight determination

#### 2.5.1. Standardization of indicator data

There are differences in data characteristics, orders of magnitude and dimensions among the indicators. In order to ensure the comparability of the indicators, this study used the range method to process the indicators dimensionless.

Positive indicator:


(8) $$\begin{array}{l} Y_{ij}=\frac{x_{ij}-min\left\{x_{j}\right\}}{\max\left\{x_{j}\right\}-min\left\{x_{j}\right\}} \end{array}$$

Negative indicator:


(9) $$\begin{array}{l} Y_{ij}=\frac{\max\left\{x_{j}\right\}-x_{ij}}{\max\left\{x_{j}\right\}-min\left\{x_{j}\right\}} \end{array}$$

Where, *Y*_*ij*_ represents the value of the *j*th index of the *i*th unit after normalization processing, *x*_*ij*_ represents the original value corresponding to the *j*th index of the *i*th unit, max{*x*_*j*_} represents the maximum value of the *j*th index, and *min*{*x*_*j*_} represents the minimum value of the *j*th index.

#### 2.5.2. Weight determination method

In this study, the coefficient of variation method was selected as the weight determination method. The coefficient of variation method is an objective method of weighting that directly uses the amount of information contained in each index and calculates the weight of the index. In the evaluation index system, the greater the difference in the value of the index, the more it can reflect the gap of the evaluated unit, and the greater its weight.

Coefficient of variation of index:


$$\begin{array}{l} V_{i}=\frac{\sigma_{i}}{{\overset{\bar{\;}}{x}}_{i}}\left(i=1,2,\cdots \;,n\right) \end{array}$$

where *V*_*i*_ is the coefficient of variation of the index *i*, also known as the coefficient of standard deviation. σ_*i*_ is the standard deviation of index *i*. ${\overset{\bar{\;}}{x}}_{i}$ is the average of index *i*.

Standard deviation of index:


$$\begin{array}{l} \sigma_{i}=\sqrt{\frac{1}{n}\sum_{i=1}^{n}{\left(x_{i}-{\bar{x}}_{i}\right)}^{2}} \end{array}$$

Weight of indicators:


(10) $$\begin{array}{l} W_{i}=\frac{V_{i}}{\sum_{i=1}^{n}V} \end{array}$$

## 3. Indicator system

According to the existing research results, the carrying capacity of coastal ecosystem is mainly affected by the three aspects of economic contribution, ecological services and human activities, and can be regarded as a composite ecosystem composed of three subsystems of economic contribution, ecological services and human activities (44, 45). The influencing factors of coastal zone economic contribution mainly include total marine economy, marine economic structure and marine economic output (46–50); The influencing factors of coastal zone ecological services mainly include biodiversity, ecological quality, life support and pollution carrying capacity (51–54); The influencing factors of human activities related to the sea mainly include population status, environmental pollution, environmental governance, residents' life, basic medical and health conditions (55–58). Therefore, the evaluation index system of coastal ecosystem carrying capacity is constructed in this study, as shown in Table 5.

**Table 5:** Evaluation index system of coastal ecosystem carrying capacity.

**Subsystem**	**Influence factor**	**Index**	**References**
Economic contribution	Economic aggregate	Gross marine product (X_1_)	(51, 59, 60)
		Per capita gross marine product (X_2_)	(46, 47, 60)
		Proportion of marine GDP in regional GDP (X_3_)	(51, 59, 60)
	Economic structure	Proportion of marine secondary industry in marine GDP (X_4_)	(47, 60, 61)
		Proportion of marine tertiary industry in marine GDP (X_5_)	(47, 60, 61)
	Economic output	Production quantity of offshore crude oil (X_6_)	(60, 62, 63)
		Production quantity of offshore natural gas (X_7_)	(60, 63, 64)
		Marine mining output (X_8_)	(53, 65)
		Output of sea salt (X_9_)	(62, 64, 66)
		Output of marine chemical products (X_10_)	(52, 53, 67)
		Cargo throughput of coastal ports (X_11_)	(53, 62, 66)
		Ocean freight volume (X_12_)	(54, 66, 67)
		Marine passenger volume (X_13_)	(48, 54, 68)
Ecological services	Bio-diversity	Phytoplankton diversity index (Y_1_)	(54, 69, 70)
		Macrozooplankton diversity index (Y_2_)	(54, 69, 70)
		Benthic diversity index (Y_3_)	(54, 69, 70)
	Ecological quality	Area of Marine Nature Reserve (Y_4_)	(53, 67, 71)
		Offshore and coastal wetland area (Y_5_)	(67, 71)
		Mariculture area (Y_6_)	(53, 60, 71)
		Proportion of Natural Reserve Area (Y_7_)	(48, 50, 55)
		Proportion of good days in bathing beach (Y_8_)	(56, 69, 72)
		Area of marine ecological monitoring area (Y_9_)	(73, 74)
	Life support	Output of seawater products (Y_10_)	(48, 49, 65)
		Number of domestic tourists in coastal cities (Y_11_)	(53, 55, 62)
	Pollution receiving capacity	Total amount of sewage discharged directly to the sea (Y_12_)	(52, 53, 57)
		Total amount of pollutants directly discharged into the sea (Y_13_)	(53, 75)
Human activity	Population status	Population density (Z_1_)	(47, 51, 53)
		Natural growth rate of population (Z_2_)	(51, 53, 76)
	Environmental pollution	Proportion of class IV and inferior class IV seawater (Z_3_)	(65, 72, 77)
		Average density of beach garbage in the monitoring area (Z_4_)	(57, 74, 78)
	Environmental governance	Total annual investment of pollution control projects (Z_5_)	(65, 78, 79)
		Proportion of environmental protection investment in GDP (Z_6_)	(51–53)
		Treatment capacity of industrial wastewater facilities (Z_7_)	(67, 69, 80)
		Comprehensive utilization quantity of industrial solid waste (Z_8_)	(62, 69, 81)
	Resident life	Per capita disposable income of urban residents (Z_9_)	(47, 51, 56)
		Per capita consumption of urban residents (Z_10_)	(47, 56, 81)
	Basic medical and health conditions	Number of medical and health institutions (Z_11_)	(58, 76)
		Number of health personnel (Z_12_)	(58, 76)
		Number of beds in medical and health institutions (Z_13_)	(58, 82)

## 4. Empirical research

### 4.1. Research area and data source

#### 4.1.1. Research area

The study area was selected as Shandong Province of China. Shandong Province is bordered by the Bohai Sea in the north and the Yellow Sea in the East. The offshore sea area accounts for 37% of the total area of the Yellow Sea and the Bohai Sea. The total length of the coastline is 3,345 km, accounting for one sixth of the length of the coastline in the whole country. Its location advantages make it an important coastal province in China and an important node of China's Maritime Silk Road (83). Shandong Province has superior geographical conditions and abundant marine resources. It has a vast sea area of about 159,500 km^2^. It is rich in beach and wetland resources. The number of high-quality beaches ranks among the top in the country. There are many high-quality islands and bays, including 589 islands and more than 200 bays. The spatial resources are extremely rich. There are more than 400 species of marine organisms with economic value (84). The advantages of marine biological resources are obvious. In terms of energy and mineral resources, the degree of abundance ranks first in China. The development potential of tidal energy, wind energy and other new marine energy is huge. There are 53 proven reserves of marine mineral resources in the province, of which nine are among the top three in China. Shandong has obvious advantages in marine resources and environment, and has great potential for marine development. From 2007 to 2020, Shandong's marine production value has always been the second highest in China (85).

Shandong Province is a large province with abundant coastal resources, and has many unique resource advantages. However, with the development of marine economy, problems such as pollution and destruction of marine ecological environment, frequent occurrence of marine disasters, and destruction of marine ecological resources and structures in the process of coastal zone development have emerged, which restrict the healthy and sustainable development of coastal zone ecosystem in Shandong Province. Therefore, it is imperative to diagnose and protect the health of coastal ecosystem in Shandong Province.

#### 4.1.2. Data source

The basic data of the empirical study are from the China Marine Statistical Yearbook (https://data.cnki.net/yearbook/Single/N2022090025), the Shandong Provincial Marine Ecological Environment Status Bulletin (http://www.sdein.gov.cn/hysthjc/gzxx/index_1.html), the China Environmental Statistical Yearbook (https://navi.cnki.net/knavi/yearbooks/YHJSD/detail), the China Health Statistical Yearbook (https://navi.cnki.net/knavi/yearbooks/YSIFE/detail?uniplatform=NZKPT), the China Environmental Quality Report (http://www.cnemc.cn/jcbg/zghjzkgb/), the China Coastal Waters Environmental Quality Bulletin (https://www.cnemc.cn/jcbg/zgjahyhjzlgb/), the China Marine Economic Statistical Bulletin (https://www.nmdis.org.cn/hygb/zghyjjtjgb/), the Shandong Statistical Yearbook (http://tjj.shandong.gov.cn/col/col6279/index.html), the Shandong Provincial Statistical Bulletin of National Economic and Social Development (http://tjj.shandong.gov.cn/col/col6196/index.html), the official website of Shandong Provincial Oceanic Administration (http://hyj.shandong.gov.cn/), the official website of Shandong Provincial Department of Ecological Environment (http://sthj.shandong.gov.cn/), etc. The data release time is 2007–2020. In this study, the data with changed statistical caliber were adjusted, and the exponential smoothing method was used to supplement a small number of missing data.

### 4.2. Data preprocessing and indicator weight

#### 4.2.1. Data preprocessing

In this study, the range method was used for dimensionless treatment of indicators, and the calculation formula is shown in Formula 8 and 9.

#### 4.2.2. Index weight

In this study, the coefficient of variation method was selected as the weight determination method, and its calculation formula is shown in Formula 10. According to the original data of the measurement indicators, the weight of each measurement indicator was calculated by the coefficient of variation method, as shown in Table 6.

**Table 6:** Index weight of coastal ecosystem carrying capacity evaluation.

**Economic contribution**		**Ecological services**		**Human activity**	
**Index**	**Weight**	**Index**	**Weight**	**Index**	**Weight**
X_1_	0.121	Y_1_	0.050	Z_1_	0.006
X_2_	0.114	Y_2_	0.091	Z_2_	0.081
X_3_	0.019	Y_3_	0.035	Z_3_	0.188
X_4_	0.028	Y_4_	0.141	Z_4_	0.169
X_5_	0.033	Y_5_	0.102	Z_5_	0.084
X_6_	0.045	Y_6_	0.046	Z_6_	0.024
X_7_	0.050	Y_7_	0.057	Z_7_	0.027
X_8_	0.179	Y_8_	0.021	Z_8_	0.055
X_9_	0.062	Y_9_	0.050	Z_9_	0.081
X_10_	0.126	Y_10_	0.036	Z_10_	0.077
X_11_	0.104	Y_11_	0.157	Z_11_	0.087
X_12_	0.060	Y_12_	0.101	Z_12_	0.062
X_13_	0.058	Y_13_	0.113	Z_13_	0.059

### 4.3. Carrying status of coastal ecosystem subsystem

In this study, the average level of indicators in the study period was taken as the evaluation standard, and the average value of indicators was taken as its ideal value (1, 43, 86), and ECC and ECC^*^ were calculated according to Formula 1.

The change of the carrying capacity of the coastal ecosystem is the result of the coupling and interaction of the three subsystems of the coastal economy, ecology and human activities. Therefore, it is necessary to analyze the three subsystems, find out the key points that restrict the level of carrying capacity of the coastal ecosystem, and respond in time to maintain the sustainable development capacity of the coastal ecosystem.

The actual value, ideal value and carrying type of the economic contribution subsystem, ecological service subsystem and human activity subsystem of Shandong Province coastal ecosystem from 2007 to 2019 are shown in Table 7.

**Table 7:** Subsystem carrying status of coastal ecosystem in Shandong Province from 2007 to 2019.

	**Year**	**2007**	**2008**	**2009**	**2010**	**2011**	**2012**	**2013**	**2014**	**2015**	**2016**	**2017**	**2018**	**2019**
Economic contribution	ECCec	0.030	0.027	0.034	0.037	0.047	0.055	0.070	0.082	0.072	0.070	0.073	0.071	0.065
	ECC*ec	**0.0504**												
	Carrying type	Light load	Light load	Light load	Light load	Loadable	Critical overload (↗)	Overload (↗)	Severe overload	Severe overload	Overload (↘)	Severe overload	Overload (↘)	Overload (↘)
Ecological services	ECCec	0.045	0.062	0.050	0.053	0.051	0.056	0.050	0.051	0.052	0.050	0.056	0.062	0.052
	ECC*es	**0.0461**												
	Carrying type	Light load	Overload (↗)	Critical overload (↘)	Critical overload (↗)	Critical overload (↘)	Overload (↗)	Critical overload (↘)	Critical overload (↗)	Critical overload (↗)	Critical overload (↘)	Overload (↗)	Overload (↗)	Critical overload (↘)
Human activity	ECCha	0.074	0.078	0.076	0.086	0.106	0.072	0.084	0.098	0.092	0.107	0.091	0.098	0.100
	ECC*ha	**0.0795**												
	Carrying type	Loadable	Loadable	Loadable	Critical overload (↗)	Overload (↗)	Loadable	Critical overload (↗)	Overload (↗)	Critical overload (↘)	Overload (↗)	Critical overload (↘)	Overload (↗)	Overload (↗)

The green arrow represents “slow” and the red arrow represents “accelerated”.

#### 4.3.1. Carrying status of coastal economic contribution subsystem

**Figure 4:**
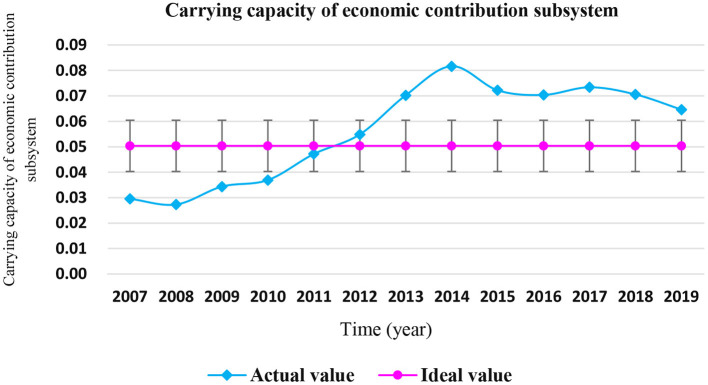
Carrying status of coastal economic contribution subsystem in Shandong Province from 2007 to 2019.

It can be seen from Table 7 that the actual values of the carrying capacity of the coastal economic contribution subsystem from 2007 to 2011 were lower than the ideal value, and the actual values were higher than the ideal values from 2012. It is particularly noteworthy that the actual values of the coastal zone economic contribution subsystem from 2013 to 2019 had always been >20% limit of the ideal values, and were in the “overload” and “serious overload” states, as shown in Figure 4.

**Figure 5:**
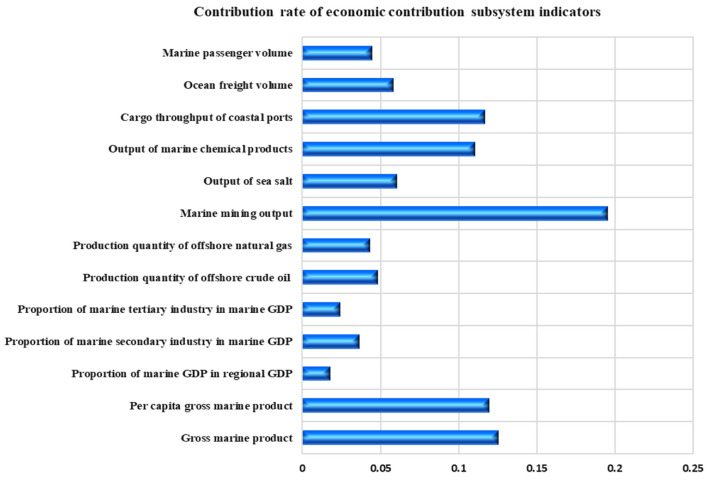
Indicator contribution rate of coastal economic contribution subsystem in Shandong Province.

The contribution rate of various indicators of the coastal economic contribution subsystem is shown in Figure 5. It can be seen that the top three indicators of contribution rate are the “marine mining output,” “marine GDP,” and “per capita marine production,” with the contribution rates of 19.52, 12.54, and 11.95%, respectively. These indicators have a great impact on the carrying capacity of the marine economic contribution subsystem of Shandong Province, and are key influencing factors.

It can be seen that Shandong Province pursues the goal of maximizing marine economic benefits, resulting in the rapid growth of marine economy. From the perspective of the output of marine economic activities in Shandong Province, the pressure of various marine economic activities on the coastal ecosystem has increased in recent years. However, since Shandong Province fully implemented the intensive utilization of sea areas in 2014, the marine industrial structure has been adjusted and optimized, and the utilization of marine resources has shown a positive development trend. The rational allocation of resources and the optimization of utilization structure have to some extent weakened the ecological pressure and negative impact of the coastal zone caused by the sea use activities.

#### 4.3.2. Carrying status of coastal ecological service subsystem

**Figure 6:**
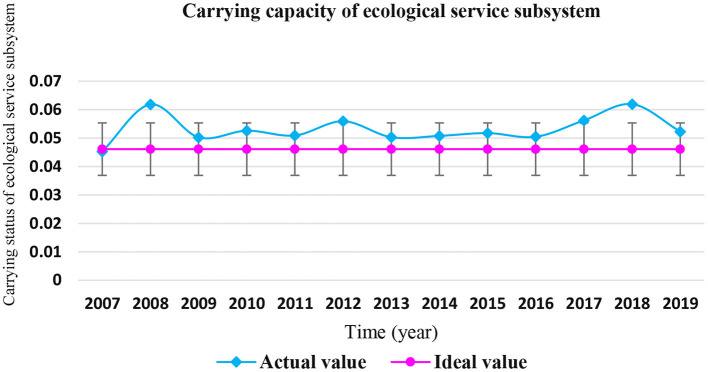
Carrying status of coastal ecological service subsystem in Shandong Province from 2007 to 2019.

It can be seen from Table 7 that the actual values of the coastal zone ecological service subsystem in Shandong Province were higher than the ideal value from 2008 to 2019. In 2008, 2012, and 2018, the actual values of the carrying capacity of the coastal ecological service subsystem exceeded the ideal value by more than 20%, and were in the “overload” state. The carrying status of coastal ecological service subsystem in Shandong Province from 2007 to 2019 is shown in Figure 6.

**Figure 7:**
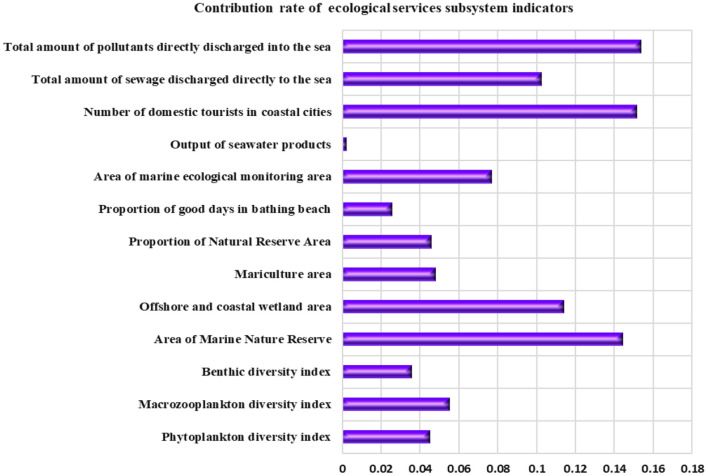
Index contribution rate of coastal ecological service subsystem in Shandong Province.

The contribution rate of various indicators of the coastal ecological service subsystem is shown in Figure 7. It can be seen that the top three indicators of contribution rate are the “total amount of pollutants directly discharged into the sea,” “number of domestic tourists in coastal cities,” and “area of Marine Nature Reserves,” with the contribution rates of 15.37, 15.16, and 14.42%, respectively. These indicators have a great impact on the carrying capacity of the marine ecological service subsystem in Shandong Province, and are key influencing factors.

The rapid development of the marine economy in Shandong Province has brought greater pressure to the coastal ecological environment, while the marine economy is still based on the extensive economic growth mode, which consumes a large amount of marine resources at the expense of the marine ecological environment, making the recovery ratio of the marine ecological service subsystem slow, resulting in a decline in the self-recovery ability of the marine ecosystem, and causing the sea area of Shandong Province to be in a state of “critical overload” and “overload.” Therefore, the key to future development is to focus on the protection and restoration of the marine ecological environment, and pursue an appropriate growth rate under the condition of coordinated resources, environment and economic and social development to achieve efficient development.

#### 4.3.3. Carrying status of coastal human activities subsystem

**Figure 8:**
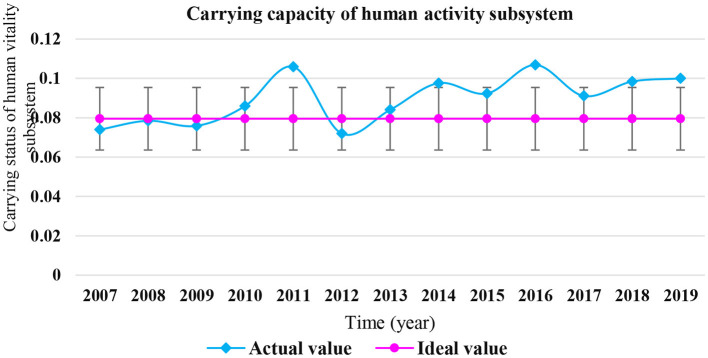
Carrying status of coastal human vitality subsystem in Shandong Province from 2007 to 2019.

It can be seen from Table 7 that from 2007 to 2010, the actual value of the carrying capacity of the human activity subsystem fluctuated up and down in the ideal value, and was in the “loadable” or “critical” state. In 2011, 2014, 2016, 2018, 2018, and 2019, the carrying capacity of the human activity subsystem were 20% higher than the ideal value, and which were in the “overload” state. There were two peaks in 2011 and 2014, respectively. The carrying status of coastal human vitality subsystem in Shandong Province from 2007 to 2019 is shown in Figure 8.

**Figure 9:**
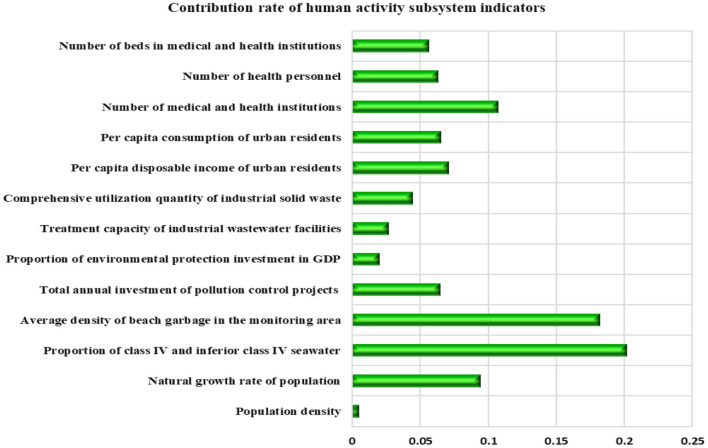
Index contribution rate of coastal human activities subsystem in Shandong Province.

The contribution rate of various indicators of the coastal human activity subsystem is shown in Figure 9. It can be seen that the top three indicators of contribution rate are the “proportion of class IV and inferior class IV seawater”, “average density of beach garbage in the monitoring area,” and “number of medical and health institutions,” with the contribution rates of 20.15, 18.22, and 10.70%, respectively. These indicators have a great impact on the carrying capacity of the marine human activity subsystem in Shandong Province, and are key influencing factors.

From the previous analysis, it can be seen that the carrying capacity of the human activities subsystem in Shandong Province accounts for the largest proportion in the carrying capacity of the marine ecosystem, while in the carrying capacity of human activities subsystem, the “proportion of seawater of class IV and inferior to class IV” and “average density of beach debris in the monitoring area” have the largest contribution rates, accounting for 38.37% in total. It shows that the coastal area pollution caused by human activities in Shandong Province has brought great pressure to the coastal ecosystem.

### 4.4. Carrying capacity of coastal ecosystem

The ecological carrying capacity of the coastal zone of Shandong Province from 2007 to 2019 is shown in Table 8.

**Table 8:** Coastal ecological carrying capacity in Shandong Province from 2007 to 2019.

**Year**	**2007**	**2008**	**2009**	**2010**	**2011**	**2012**	**2013**	**2014**	**2015**	**2016**	**2017**	**2018**	**2019**
ECC	0.092	0.104	0.097	0.107	0.127	0.106	0.121	0.137	0.128	0.138	0.130	0.136	0.130
ECC^*^	**0.1048**												
Carrying type	Loadable	Loadable	Loadable	Critical overload (↗)	Critical overload (↗)	Critical overload (↘)	Critical overload (↗)	Overload (↗)	Overload (↘)	Overload (↗)	Overload (↘)	Overload (↗)	Overload (↘)

The green arrow represents “slow” and the red arrow represents “accelerated”.

**Figure 10:**
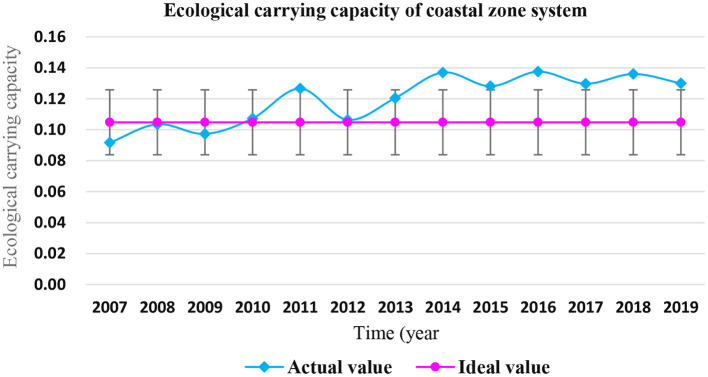
Carrying capacity of coastal ecosystem in Shandong Province from 2007 to 2019.

It can be seen from Figure 10 that the carrying capacity of the coastal ecosystem in Shandong Province was relatively good from 2007 to 2009, and the actual carrying capacity ECC was less than the ideal value ECC^*^, which was in a “loadable” state. From 2010 to 2013, the actual carrying capacity ECC was not >20% of the ideal state value ECC^*^, which was in the “critical overload” state. From 2014 to 2019, the carrying capacity of the coastal ecosystem in Shandong Province was poor, the actual carrying capacity ECC was 20% higher than the ideal value ECC^*^, which was in the “overload” state, the carrying capacity of the coastal ecosystem showed a “fluctuating overload” trend. It can be seen that the carrying capacity of Shandong coastal ecosystem is not optimistic in recent years.

**Figure 11:**
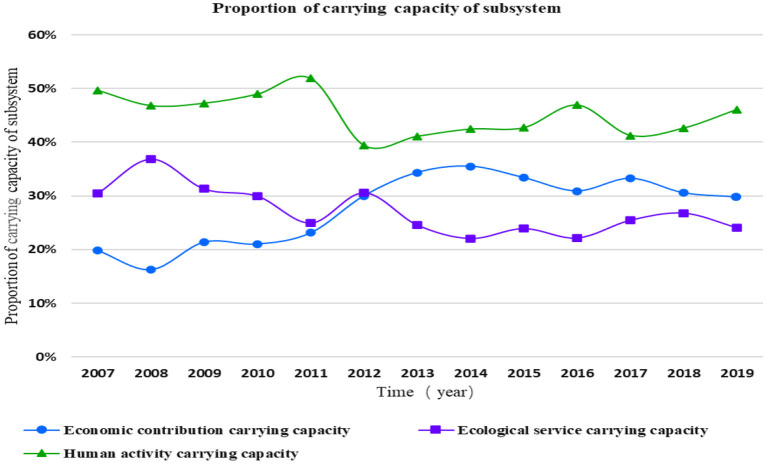
Carrying capacity of coastal ecosystem subsystem in Shandong Province from 2007 to 2019.

The carrying status of marine ecosystem in Shandong Province from 2007 to 2019 is shown in Figure 11. From 2007 to 2019, the carrying capacity of the human activity subsystem of the coastal ecosystem of Shandong Province was at a high level, accounting for the largest proportion of the carrying capacity of the marine ecosystem of Shandong Province, accounting for 45% on average.

The carrying capacity of the economic contribution subsystem was lower than that of the ecosystem service subsystem from 2007 to 2012, and higher than that of the ecosystem service subsystem from 2013 to 2019. Therefore, the carrying capacity of the economic contribution subsystem and the ecological service subsystem of Shandong Province accounted for 28 and 27% of the marine ecosystem carrying capacity of Shandong Province, respectively. It can be seen that the human activity subsystem in Shandong Province had a great impact on the carrying capacity of the marine ecosystem from 2007 to 2019.

### 4.5. Coastal ecosystem health diagnosis

Based on the above analysis of the coastal ecosystem carrying status, according to the coastal ecosystem health diagnosis framework of “Carrying type → Early warning degree → Health level,” the coastal ecosystem health level of Shandong Province from 2007 to 2019 can be obtained, as shown in Table 9.

**Table 9:** Health level of coastal ecosystem in Shandong Province from 2007 to 2019.

**Year**	**2007**	**2008**	**2009**	**2010**	**2011**	**2012**	**2013**	**2014**	**2015**	**2016**	**2017**	**2018**	**2019**
Carrying status ↓	0.092	0.104	0.097	0.107	0.127	0.106	0.121	0.137	0.128	0.138	0.130	0.136	0.130
Carrying type ↓	Loadable	Loadable	Loadable	Critical overload (↗)	Critical overload (↗)	Critical overload (↘)	Critical overload (↗)	Overload (↗)	Overload (↘)	Overload (↗)	Overload (↘)	Overload (↗)	Overload (↘)
Change trend ↓	—	—	—	↗	↗	↘	↗	↗	↘	↗	↘	↗	↘
Early warning level ↓	Green warning	Green warning	Green warning	Yellow warning	Yellow warning	Blue warning	Yellow warning	Red warning	Orange warning	Red warning	Orange warning	Red warning	Orange warning
Early warning degree ↓	No warning	No warning	No warning	Medium warning	Medium warning	Light warning	Medium warning	Extremely heavy warning	Heavy warning	Extremely heavy warning	Heavy warning	Extremely heavy warning	Heavy warning
Health level ↓	Very healthy	Very healthy	Very healthy	Sub-health	Sub-health	Health	Sub-health	Morbid	Unhealthy	Morbid	Unhealthy	Morbid	Unhealthy

The green arrow represents “slow” and the red arrow represents “accelerated”.

### 4.6. Empirical conclusion

(1) The coastal ecosystem of Shandong Province has been “overloaded” state in recent years

From 2007 to 2013, the carrying capacity of the coastal ecosystem in Shandong Province was relatively good, in the state of “loadable” or “critical overload,” while from 2014 to 2019, the carrying capacity was poor, in the state of “overload.”

(2) The health of coastal ecosystem in Shandong Province has been “unhealthy” or “morbid” state in recent years

From 2007 to 2013, the early warning degree of coastal ecosystem health in Shandong Province was in the state of “no alarm,” “light alarm,” and “medium alarm,” and the health level was in the state of “very healthy,” “healthy,” and “sub-health”; from 2014 to 2019, the health warning level of the coastal ecosystem in Shandong Province was in the state of “serious alarm” and “extremely serious alarm,” and the health level was in the state of “unhealthy” and “morbid,” and the health status was worrying.

(3) Key influencing factors of coastal ecosystem carrying capacity in Shandong Province

The key influencing factors affecting the carrying capacity of the coastal ecosystem mainly include the “output of marine mining,” “marine GDP,” “per capita marine production,” “total amount of main pollutants directly discharged into the sea,” “domestic tourist arrivals in coastal cities,” “area of marine nature reserves,” “proportion of class IV and worse than class IV seawater,” “average density of beach garbage in the monitoring area,” and “number of medical and health institutions.”

### 4.7. Coping strategies

According to the health diagnosis results of the coastal ecosystem in Shandong Province, the coastal ecosystem in Shandong Province is in a “morbid” state in recent years, which needs to be paid enough attention and effective corresponding strategies should be taken.

#### 4.7.1. Real time monitoring and early warning

Strengthen the control of pollution in alongshore sea area and key bays of Shandong Province, establish a long-term and standardized marine monitoring system, and conduct real-time monitoring on the main rivers and sewage outlets entering the sea; The indicators of ecological carrying capacity overload and critical overload in the coastal zone shall be given early warning, and the main pressure and driving force leading to the overload or critical overload of the indicator and the main control force that may improve the overload condition of the indicator shall be comprehensively analyzed. Through strict assessment of the water quality of the river sections entering the sea, strictly control the discharge of pollutants into the sea in Laizhou Bay, Jiaozhou Bay, and Xiaoqing River, strictly control the construction projects of the sea related projects that discharge pollutants into the sea, try to strengthen pollution control and habitat restoration, and appropriately reduce the number of new sewage outlets and sewage plants that discharge into the sea. The database and decision support system shall be established in the aspects of overall sea area development intensity control, total amount control of pollutants, ecological environment remediation and protection, coping with climate change and environmental disaster risk, and gradually realize that each decision is supported by scientific data.

#### 4.7.2. Total emission control

Strictly control the total amount of pollution emissions from coastal industries and industrial clusters; for the newly added reclamation area, the sea use planning shall be approved according to the regional emission reduction standards; Increase the monitoring indicators of sewage discharge within a reasonable range, rectify and eliminate backward and seriously polluting enterprises related to the sea, and increase funds to support enterprises with low energy consumption, low pollution and high efficiency; Optimize the layout of the mariculture industry, actively improve the technical measures for aquaculture, use environmentally friendly feed, strictly control the amount of aquaculture drugs, and effectively control the total amount of nitrogen and phosphorus discharged into the sea; promote the construction of pollution-free marine aquaculture bases, standardize aquaculture methods, strictly control the use of aquaculture feed and drugs, and vigorously develop circular economy and ecological aquaculture; improve the treatment capacity of polluted wastes from ports and ships, control the floating pollution at sea, strengthen the effective supervision of ports, ships, offshore drilling, and other related operations, and put an end to oil spill accidents and illegal dumping of wastes (53).

#### 4.7.3. Overall planning of land and sea

The coastal zone ecosystem is at the junction of the marine ecosystem and the terrestrial ecosystem. It is necessary to promote the land and sea coordination, strengthen the top-level design, strictly formulate the marine protection and comprehensive utilization planning of marine resources, and attach importance to the protection and restoration of the land and sea natural ecosystems in the coastal zone (87). From the perspective of ocean, it is necessary to strictly control reclamation activities, carry out construction in strict accordance with the development plan, and avoid unrestrained and simple pursuit of economic output; protect wetlands and tidal flats, formulate strict laws and regulations, and ensure the implementation of the coastal protection mechanism. From the perspective of land, control the discharge of pollution sources, improve the sewage treatment capacity and garbage treatment capacity, limit the pollution discharge of coastal industrial production, eliminate backward production capacity, and optimize the layout of marine related industries. According to the capacity of the marine environment, the limited standards for pollutant discharge in different industries, coastal areas and inland areas shall be formulated, and the total amount of pollutant discharge shall be controlled.

#### 4.7.4. Intensive use of sea area

The intensive utilization of the sea area can further optimize the project design of the intensive utilization on the basis of land and sea coordination, accelerate the improvement of the utilization efficiency of the sea area resources, speed up the combination degree of the intensive utilization and environmental remediation, further improve the level of marine ecological environment governance and management, give full play to the advantages of the intensive utilization of the sea area, enhance the ecological carrying capacity, and protect the marine ecological functions. Optimize the marine industry through economic regulation and control, implement sewage charges, formulate a high standard and perfect system of intensive use of sea areas and standardized management, accelerate the adjustment of the marine industrial structure and change the mode of economic growth, so as to expand economic benefits and relieve ecological pressure.

#### 4.7.5. Strictly observe the ecological red line

Strictly control the development and utilization activities that affect the ecological functions of the coastal ecosystem, and delimit the ecological red line of the sea area, especially the important marine ecological areas, marine ecological functional areas and marine ecological vulnerable areas. According to the temporal and spatial development trend of coastal ecosystem, reasonably regulate the marine ecological protection red line, so as to improve the marine ecological function, ensure the stability of marine ecological subsystem, and balance the relationship between coastal ecosystem function and marine economic and social development (45). Strictly implement the ecological red line system of the whole sea area, and bring all marine protected areas, important coastal wetlands, and important sandy shorelines under the control of the ecological red line area to effectively protect the ecological environment of coastal zone.

#### 4.7.6. Actively advocate public participation

Strengthen the guidance for citizens to participate in marine protection activities, and introduce corresponding policies and systems to protect citizens' rights to freely participate in marine health activities. At the institutional and organizational levels, it provides guarantee for marine social organizations to participate in marine affairs management. Under the background of policy guarantee, emphasize the voluntary equality and cooperation among all social subjects, and mobilize the whole society to implement large-scale, diversified and multi-level capital investment for coastal ecosystem protection (45). Improving public awareness of coastal ecosystem services and marine ecological environment carrying capacity is conducive to promoting the connection between science and management (88). In order to facilitate the realization of this connection, relevant education policies should be issued for coastal ecosystem, such as increasing marine education investment policies, setting multi-level and multi-field marine education policies, and giving appropriate priority to the allocation of marine education resources. Initiating citizen science could help to develop a science educated society that will respond more positively to better environmental management (89).

## 5. Discussion

### 5.1. The consistency between the empirical research results and the development status of the research region

Since 2008, Shandong Province has implemented the strategy of “vigorously developing the marine economy and building a strong marine province.” From 2009 to 2011, Shandong Province has successively been approved with various development plans such as the “Development Plan of the Efficient Ecological Economic Zone in the Yellow River Delta” and the “Development Plan of the Shandong Peninsula Blue Economic Zone.” The development of marine resources is hot, the marine economy is developing rapidly, and the gross marine product has rapidly increased from 534.63 billion yuan in 2008 to 1,480 billion yuan in 2019, it has always ranked second in the country. The marine economy is dominated by extensive growth and consumes a large number of marine resources at the expense of marine ecological environment. The rapid development of marine economy has brought enormous pressure to the marine resources and environment system, which has aggravated the consumption of marine resources and the deterioration of marine ecological environment. Due to the inability to curb the negative impact of human development and utilization and social and economic development on the coastal ecosystem, and the unscientific use of sea areas, the coastal ecological carrying capacity fluctuated sharply from 2009 to 2013. The resilience of marine resources and environment, which is the main influencing factor of the carrying capacity of the coastal zone ecosystem. Due to the rapid development of marine resources, the recovery of marine resources and environmental systems lags far behind the development of marine resources, which leads to insufficient self-recovery capacity of coastal ecosystems and overloading of coastal ecosystems in Shandong Province in recent years. In recent years, Shandong Province has preliminarily established the marine environment monitoring and evaluation business system of the whole province, and basically established the administrative classification and business guidance type marine environment monitoring and network operation mechanism. A total of 34 marine environment monitoring institutions are set up in the province, which are composed of provincial, municipal and county level monitoring institutions. The facilities and funds basically meet the needs of assessment and early warning. Ecological carrying capacity assessment and early warning are conducted by feeding back the monitoring data of various indicators in each year, thus guiding Shandong Province to carry out coastal zone ecological protection under the intensive use of sea areas. However, due to the slow recovery of marine resources and environment, the ecological carrying capacity of Shandong Province coastal zone is still in a slow state of adjustment and improvement. Therefore, the key to future development is to focus on marine environment protection and restoration, pursue moderate growth rate under the condition of coordinated resources, environment and economic and social development, and achieve efficient development.

It can be seen that the empirical research conclusion of this paper is basically consistent with the actual situation of the development of the marine economy, ecology and social complex system in Shandong Province.

### 5.2. The consistency between the research conclusions of this paper and the conclusions of existing research results

The relevant studies on marine ecological carrying capacity in Shandong Province are shown in Table 10.

**Table 10:** Related research on marine ecological carrying capacity in Shandong Province.

	**Existing research results**	**Methods adopted**	**Main research conclusions**
I	Study on evaluation of marine resources and environmental carrying capacity in Shandong province (90)	Entropy weighted- TOPSIS model	From 2007 to 2017, the carrying capacity of marine resources and environment in Shandong Province showed an obvious upward trend.
II	Evaluation and early warning of sea area carrying capacity in Shandong Province based on multidimensional state space and neural network model (91)	Multidimensional state space method and BP neural network method	Under the scenario of “high development” of resources, environment, economy and society subsystems, sea area of Shandong Province will reach a state of weak sustainable development in 2015.
III	Comprehensive evaluation of sustainable development capacity of marine ecology in Shandong Peninsula Blue Economic Zone based on DPSIR model (92)	DPSIR model	The social and economic pressure on Shandong Peninsula is increasing. The comprehensive assessment value of marine ecosystem is 0.53, and the current situation of marine ecological environment is not optimistic.
IV	Evaluation of marine ecological environment based on Bayesian Network–A case study of Shandong Province (80)	Bayesian network method	From 2013 to 2019, the coastal ecological environment of Shandong Province remained in a general state, and the indicators of marine environmental quality and marine ecological disasters had the greatest impact on the assessment results.
V	Evaluation of the sea area carrying capacity of Shandong Peninsula Blue Economic Zone (93)	Mean square error analytical method	The pressure index of Shandong Peninsula Blue Economic Zone shows a general downward trend, the carrying capacity index shows an overall upward trend, and the pressure ratio shows an overall upward trend.
VI	Assessment of marine ecological carrying capacity in Shandong Province based on SAD-SAS model (94)	Improved SAD-SAS model	From 2006 to 2018, the level of marine ecological carrying capacity in Shandong Province generally maintained an upward trend, but there were large fluctuations.

Therefore, it is obvious that the conclusions of this study are consistent with those of previous studies to some extent.

### 5.3. Limitations and applicability of the research methods in this paper

The diagnosis method in this paper can diagnose the health status of coastal ecosystem according to certain steps and levels, the analysis framework is clear, progressive and linked, and has strong logic and practicality. However, it has greater flexibility in determining the ideal value standard of carrying capacity. For example, the ideal value can be determined according to the average value of the data in the research period (commonly used), the ideal value can be determined according to the maximum value of the data in the research period, the ideal value can be determined according to the standards specified by the industry, and the ideal value can also be determined according to the planning objectives. Due to different determination methods of the ideal value standard of bearing capacity, different diagnosis results may be obtained, and there may be some human factors in the diagnosis.

Therefore, the diagnosis method in this paper is more suitable for comparative analysis under the same ideal value standard. For example, (1) For diagnosis of the same ecosystem. The management department conducts comparative analysis on the health status of the same ecosystem in different years (such as the example in this article) to find out the insufficient management; (2) It is used to evaluate the management performance of different ecosystems. Evaluate the management performance of the ecosystem management department according to the same standards to put forward more specific work requirements; (3) It is used for the assessment of ecosystem health in different regions. Evaluate ecosystem health in different regions according to the same evaluation criteria to reward the good and punish the bad. When determining the ideal value of bearing capacity, the standard can be selected according to the specific needs or work requirements.

## 6. Conclusion

In order to maintain the long-term stability of the coastal ecosystem function, it is necessary to artificially provide external support to maintain the basic balance of the comprehensive carrying capacity of the coastal zone ecosystem. Only on the premise of long-term loadable state of coastal ecosystems, human social and economic activities can continue. The coastal ecosystem health diagnosis not only plays an important guiding role in the early stage of the coastal zone resources development and utilization planning, but also needs to be carried out at any stage of the planning implementation. It is very important to master the environmental capacity and carrying capacity of ecological resources in the coastal zone. For those exceeding the limit of ecological resources and environmental carrying capacity of the coastal zone, it is necessary to resolutely take measures as soon as possible to reduce the carrying pressure of resources and environment, and take reasonable and effective repair measures according to the specific situation of health diagnosis, so as to prevent greater irreparable damage to ecological resources and environment. Attach great importance to those that are still within the carrying capacity. Do not wait until the ecological red line is touched and the resources and environment are damaged before repairing, do not pay an undue price. Always regard the health diagnosis of coastal ecosystem as the limiting standard for marine development and utilization, not exceeding the carrying capacity of resources and environment, scientifically and rationally develop and utilize coastal resources, and effectively maintain the health and sustainable development of coastal ecosystem.

## Data availability statement

Publicly available datasets were analyzed in this study. This data can be found at: the China Marine Statistical Yearbook, the Shandong Provincial Marine Ecological Environment Status Bulletin, the China Environmental Statistical Yearbook, the China Health Statistical Yearbook, the China Environmental Quality Report, the China Coastal Waters Environmental Quality Bulletin, the China Marine Economic Statistical Bulletin, the Shandong Statistical Yearbook, and the Shandong Provincial Statistical Bulletin of National Economic and Social Development.

## Author contributions

CZ: data curation, methodology, software, and original draft preparation. MW: conceptualization, improvement, and editing. Both authors contributed to the article and approved the submitted version.

## References

[1] Cui HT He GZ Lv YL Yuan JJ. Comprehensive evaluation of urban ecological carrying capacity in coastal zone. J Ecol. (2020) 40:2567–76. 10.5846/stxb20190213025735229523

[2] Lv YL Yuan JJ Li QF Zhang YQ Lv XT Su C. Effects of terrestrial human activities on coastal ecosystems. J Ecol. (2016) 36:1183–91. 10.5846/stxb201511182334

[3] Wu CL. Evaluation of coastal resources and environmental carrying capacity of Fujian Province. J Shangqiu Normal Univ. (2018) 34:46–50. 10.3969/j.issn.1672-3600.2018.03.01333601669

[4] Yang CX. A preliminary study on the evaluation of resources and environment carrying capacity of coastal zone in China. J Ocean Dev Manag. (2016) 6:109–12. 10.3969/j.issn.1005-9857.2016.06.020

[5] Ayinuer Y Zhang F Tan ML. Spatial-temporal characteristics of ecosystem health in Central Asia. Int J Appl Earth Observ Geoinformat. (2021) 105:102635. 10.1016/j.jag.2021.102635

[6] Shi RX Jia QY Wei FZ Du GZ. Comprehensive evaluation of ecosystem health in pastoral areas of Qinghai–Tibet Plateau based on multi model. J Environ Technol Innov. (2021) 23:101552. 10.1016/j.eti.2021.101552

[7] Liu Y Yang C Tan SH Zhou HT Zeng W. An approach to assess spatiotemporal heterogeneity of rural ecosystem health: A case study in Chongqing mountainous area, China. J Ecol Indicat. (2022) 136:108644. 10.1016/j.ecolind.2022.108644

[8] Tu WB Zhang XX Fu ZT. Model for selecting indicators of rural ecosystem health assessment based on multi-objective programming. J System Eng Theory Practice. (2012) 32:2229–36. 10.3969/j.issn.1000-6788.2012.10.014

[9] Wang QS Liu MQ Tian S Yuan XL Ma Q Hao HL. Evaluation and improvement path of ecosystem health for resource-based city: A case study in China. J Ecol Indicat. (2021) 128:107852. 10.1016/j.ecolind.2021.107852

[10] Shen W Zheng ZC Pan L Qin YC Li Y. A integrated method for assessing the urban ecosystem health of rapid urbanized area in China based on SFPHD framework. J Ecol Indicat. (2021) 121:107071. 10.1016/j.ecolind.2020.107071

[11] Guo RZ Shen HJ Yang MH. Study on land ecosystem health assessment in Chang-Zhu-Tan Region based on improved PSR model. J Environ Monitor Manag Technol. (2021) 33:29–34. 10.3969/j.issn.1006-2009.2021.03.007

[12] Yang YJ Song G Lu S. Assessment of land ecosystem health with Monte Carlo simulation: A case study in Qiqihaer, China. J Clean Prod. (2020) 250:119522. 10.1016/j.jclepro.2019.119522

[13] Chen XJ Li CC Huang JY Chen X. Remote sensing diagnosis of forest ecosystem health in Baili Rhododendron Forest Region. J Guizhou Prov Guizhou Sci. (2021) 39:41–7. 10.3969/j.issn.1003-6563.2021.06.011

[14] Zhou Q Ye M Zhao FF. Forest ecosystem health assessment in Altai Mountain Forest region based on VOR model. J Gansu Agri Univ. (2021) 3:137–48. 10.13432/j.cnki.jgsau.2021.03.018

[15] Zhang KL Ye M Yin XK Guo JX Zhao FF. Assessment of grassland ecosystem health in Qinghe forest region. J Gansu Agri Univ. (2022) 2:182–8. 10.13432/j.cnki.jgsau.2022.02.022

[16] Chen CB Peng J Li GY. Establishment of grassland ecosystem health assessment system in Xinjiang. J Study Arid Areas. (2022) 39:270–81. 10.13866/j.azr.2022.01.26

[17] Zhang SY Yuan ZC Li ZQ Li TH. Ecological health assessment and degradation prediction of wetland in Qinmang River basin based on PSR model. J Henan Sci. (2022) 40:1619–27.

[18] Zhang MY Wang XH Tong SZ Zhang DJ Qi Q An Y. Ecosystem health assessment of Yuehai National Wetland Park in Ningxia. J Environ Sci Technol. (2022) 45:247–53. 10.19672/j.cnki.1003-6504.0832.21.338

[19] Wu Z Chen RS Meadows Michael E Liu X. Application of the Ocean Health Index to assess ecosystem health for the coastal areas of Shanghai, China. J Ecol Indicat. (2021) 126:107650. 10.1016/j.ecolind.2021.107650

[20] Rombouts I Beaugrand G Artigas LF Dauvin JC Gevaert F Goberville E. Evaluating marine ecosystem health: Case studies of indicators using direct observations and modelling methods. J Ecol Indicat. (2013) 24:353–65. 10.1016/j.ecolind.2012.07.001

[21] Zheng T You XY. Assessment of marine ecosystem health of Tianjin Offshore, China. J Oceanol Hydrobiol Stud. (2013) 42:442–50. 10.2478/s13545-013-0100-0

[22] Joesidawati MI Suwarsih S. Measurement of indonesian marine health index to assess the health of the coastal ecosystem of Tuban, East Java. J Earth Environ Sci. (2022) 2022:1036. 10.1088/1755-1315/1036/1/012046

[23] Li QX Zhu L Chen ZZ. Evaluation of offshore marine ecosystem health by grey system method. J Nankai Univ. (2010) 43:39–43. 10.3969/j.issn.0465-7942.2010.01.009

[24] Song DB Gao ZQ Zhang H Xu FX Zheng XY Ai JQ. GIS-based health assessment of the marine ecosystem in Laizhou Bay, China. J Mar Poll Bullet. (2017) 125:242–9. 10.1016/j.marpolbul.2017.08.027 28823550

[25] Seung HB Son M Kim D Choi HW Kim YO. Assessing the ecosystem health status of Korea Gwangyang and Jinhae bays based on a planktonic index of biotic integrity (P-IBI). J Ocean Sci J. (2014) 49:291–311. 10.1007/s12601-014-0029-2

[26] Hermosillo-Núñez BB Ortiz M Rodríguez-Zaragoza FA Cupul-Magaña AL. Trophic network properties of coral ecosystems in three marine protected areas along the Mexican Pacific Coast: Assessment of systemic structure and health. J Ecol Complex. (2018) 36:73–85. 10.1016/j.ecocom.2018.06.005

[27] Ji YN Niu WT Huang DY Huang SL Wang CS. Construction and application of coral reef ecosystem health assessment index system based on PSR model. J Appl Oceanogr. (2014) 3:343–7. 10.3969/J.ISSN.2095-4972.2014.03.008

[28] Prasetya JD Ambariyanto Supriharyono Purwanti F. Hierarchical synthesis of coastal ecosystem health indicators at Karimunjawa National Marine Park. J Earth Environ Sci. (2018) 116:12094. 10.1088/1755-1315/116/1/012094

[29] Prasetya JD Ambariyanto Supriharyono Purwanti F. Mangrove health index as part of sustainable management in mangrove ecosystem at Karimunjawa National Marine Park. Indonesia J Continent Shelf Res. (2016) 121:1–2. 10.1166/asl.2017.9155

[30] Zhang H Xiao Y Deng YZ. Island ecosystem evaluation and sustainable development strategies: A case study of the Zhoushan Archipelago. J Glob Ecol Conserv. (2021) 2021:e01603. 10.1016/j.gecco.2021.e01603

[31] Wang S Li JQ Hou YW Sun JF. The construction of marine ranch ecosystem health assessment index system based on PSR model. J Hebei Fish. (2020) 4:28–60. 10.3969/j.issn.1004-6755.2020.04.010

[32] Ding JK Zhang WW Li Y Xue SY Li JQ Jiang ZJ. Assessment of benthic ecosystem health in Jiaozhou Bay based on ecological characteristics of macrobenthos. J Adv Fish Sci. (2020) 2:20–6. 10.19663/j.issn2095-9869.20181104001

[33] Zhao LL Fan XC He DL. Coastal ecosystem health assessment: A case study of Ningde Marine Ecological Special Reserve. J Anhui Agri Sci. (2020) 24:71–4. 10.3969/j.issn.0517-6611.2020.24.021

[34] Yang ZF Sui X. Evaluation of ecological carrying capacity based on ecosystem health. J Environ Sci. (2005) 25:586–94. 10.13671/j.hjkxxb.2005.05.004

[35] Di QB Zhang J Wu JL. Evaluation of marine ecological carrying capacity of Liaoning Province Based on ecosystem health. J Nat Resour. (2014) 29:256–254.

[36] Zhang J. Study on Marine Ecological Carrying Capacity – A Case Study of Liaoning Province. Dalian: Liaoning Normal University (2013). p. Y2377208.

[37] Li Y. Research on Monitoring and Early Warning Index Selection and System Construction Method of Resources and Environment Carrying Capacity Based on Marine Health. Shanghai: Shanghai Ocean University (2019).

[38] Ma PP. Study on Ecological Carrying Capacity of Coastal Zone in Zhejiang Province. Hangzhou: Zhejiang University (2017). Available online at: https://d.wanfangdata.com.cn/thesis/ChJUaGVzaXNOZXdTMjAyMzAxMTISCFkzMzA0NjczGghycHdrYnI5cw%3D%3D

[39] Li HT Gu CJ Liang T. New perspective and application of ecosystem health assessment. J Nat Resour. (2010) 25:1797–805.

[40] Di QB Han ZL. Quantitative discussion on the carrying capacity of sea area – take Liaoning sea area as an example. J Ocean Bullet. (2005) 24:48–55. 10.3969/j.issn.1001-6392.2005.01.009

[41] Xu DL Li YQ. Evaluation of marine ecological environment carrying capacity based on state space method. J Statist Decision Mak. (2014) 18:59–60. 10.13546/j.cnki.tjyjc.2013.18.01834861505

[42] Li M Dong SY Zhang HH Di QB. Research on the evaluation and early warning of sea area carrying capacity in Shandong Province Based on multidimensional state space and neural network model. J Ocean Bulletin. (2015) 34:608–15. 10.11840/j.issn.1001-6392.2015.06.002

[43] Wei C Ye SF Guo ZY Liu HQ Deng BP Liu X. Construction and application of evaluation index system of coastal zone comprehensive carrying capacity – A case study of Nantong city. J Ecol. (2013) 33:5893–904. 10.5846/stxb201304090649

[44] Wang JQ Wu JC He CY Wu JL. Ecological carrying capacity evaluation of coastal zone in Zhejiang Province Based on weight entropy method. J Zhejiang Agri Sci. (2019) 60:848–51. 10.16178/j.issn.0528-9017.20190554

[45] Zhang C Wang M. Assessing the conjugacy of the marine economy-ecology-society composite system: China's Case. J Front Mar Sci. (2022) 8:963468. 10.3389/fmars.2022.963468

[46] Liang YH. Research on the Index Evaluation System of Strong Marine Province. Dalian: Dalian Maritime University (2017). Available online at: https://d.wanfangdata.com.cn/thesis/ChJUaGVzaXNOZXdTMjAyMzAxMTISCFkzMzQ4MDcyGghneXl2aDg2ag%3D%3D

[47] Gao Q Liu T Wang Y Ding CC. Study on evaluation of coordinated development of marine eco-economic system. J Mar Environ Sci. (2019) 38:568–74. 10.13634/j.cnki.mes.2019.04.013

[48] Lu W. Evaluation of Sustainable Development of Marine Ecological Economy in Jiangsu Province. Qingdao: Ocean University of China (2014). Available online at: https://d.wanfangdata.com.cn/thesis/ChJUaGVzaXNOZXdTMjAyMzAxMTISB0Q1NDc0MjQaCGtjdW12Ymx0

[49] Zhen JH Di QB. The coupling analysis between the development level of marine economy and the carrying capacity of the Bohai Rim region. J Mar Econ. (2017) 7:37–46. 10.3969/j.issn.2095-1647.2017.05.005

[50] Liu B long RY Zhu CG Sun XX. Measuring the level of coordinated development of marine economy and ecological environment. J Explor Econ Probl. (2020) 12:55–65.

[51] Ding XY He JL. Evaluation of marine resources and environment carrying capacity in Shandong Province. J Mar Econ. (2021) 11:72–9. 10.3969/j.issn.2095-1647.2021.04.009

[52] Wang ZF Li T Gao WM. Coupling analysis of marine resources and environment carrying capacity and economic development level in coastal areas of Hebei Province. J Mar Limnol Bullet. (2021) 43:58–65. 10.13984/j.cnki.cn37-1141.2021.06.009

[53] Li H. Evaluation and Prediction of Marine Comprehensive Carrying Capacity in Zhejiang Province. Zhoushan: Zhejiang Ocean University (2020).

[54] Cao YC. Evaluation of marine resources and environment carrying capacity based on entropy weight TOPSIS Model: A case study of Zhanjiang City. J Mar Bullet. (2019) 38:266–72. 10.11840/j.issn.1001-6392.2019.03.004

[55] Zheng L Cai DH Yang N. An empirical study on marine economic evaluation system based on sustainable development. J Mar Econ. (2015) 5:21–31. 10.19426/j.cnki.cn12-1424/p.2015.01.004

[56] Zhou L You GB. Evaluation and model selection of sustainable development of marine ecological economy in Fujian Province. J Mar Econ. (2017) 7:30–7. 10.3969/j.issn.2095-1647.2017.02.005

[57] Gao Q. Study on Comprehensive Evaluation of Marine Economic Development Quality in 11 Coastal Provinces and Cities of China. Dalian: Liaoning Normal University. (2016). Available online at: https://d.wanfangdata.com.cn/thesis/ChJUaGVzaXNOZXdTMjAyMzAxMTISCFkzMTI4NjA5GghzamF6eXd4Mw%3D%3D

[58] Zhao CX. Study on comprehensive evaluation method of regional population health status. J Smart Health. (2018) 4:17–23. 10.19335/j.cnki.2096-1219.2018.28.009

[59] Wang B Chang JC. Evaluation and analysis of marine economic competitiveness in China's coastal areas. J Mar Dev Manag. (2019) 7:77–88. 10.3969/j.issn.1005-9857.2019.07.014

[60] Zhao LD Shao SY Sui HK. Study on the coupling coordination between marine industry agglomeration and marine ecological resources and environment in Shandong Province. J Oriental For. (2018) 3:29–34. 10.3969/j.issn.1005-7110.2018.03.005

[61] Liu X. Research on the Evaluation of Marine Economic Development Level in Shandong Province. Changsha: Central South University of Forestry Technology (2019). Available online at: https://d.wanfangdata.com.cn/thesis/ChJUaGVzaXNOZXdTMjAyMjA1MjYSCFkzNjEyMjUzGghyZzVza3NpaQ%3D%3D

[62] Zhang X Bai FC. Study on the coupling relationship between marine resources and environment system and marine economic system in Guangdong Province. J Ecol Econ. (2018) 34:75–80.

[63] Wang YM Di MQ Wang YC. Study on the coupling and coordinated development of marine economic composite system in Shandong Province. J Shandong Inst Bus Technol. (2021) 35:1–10. 10.3969/j.issn.1672-5956.2021.05.001

[64] Li GZ Hao TT Zhao KX. Analysis on the coupling and coordinated development of marine economy, resources and environment in China's coastal areas. J Jilin Normal Univ. (2018) 2018:81–7. 10.3969/j.issn.2096-2991.2018.02.011

[65] Gu YJ Qian LF Bie M. Jiangsu marine economy high quality development level evaluation and countermeasure research. J Mar Dev Manag. (2021) 12:32–40. 10.3969/j.issn.1005-9857.2021.12.005

[66] Wang M Di QB. Study on the coupling relationship between the carrying capacity of marine resources and the development potential of marine economy in the Bohai Rim region. J Mar Dev Manag. (2016) 1:33–9. 10.3969/j.issn.1005-9857.2016.01.005

[67] Shang Q. Research on Evaluation and Early Warning of Coordinated Development of Liaoning Marine Economic System. Dalian: Liaoning Normal University (2021).

[68] Luo M. Distribution of Enteromorpha green tide in Jiangsu province sea area and its impact on ecosystem. J Environ Dev. (2019) 31:177–9. 10.16647/j.cnki.cn15-1369/X.2019.11.100

[69] Xu ST Guan W. Evaluation on sustainable development of marine economy in Bohai Rim region. J China's Mar Econ. (2017) 2:119–34.

[70] Zhai RX. Measurement and evaluation of marine carrying capacity in Jiangsu Province. Jiangsu Agri Sci. (2014) 42:398–401. 10.15889/j.issn.1002-1302.2014.04.137

[71] Liu B Long RY Zhu CG Sun XX Pan KY. Evaluation on high quality development level of marine economy in Jiangsu Province. J Econ Geogr. (2020) 40:104–13. 10.15957/j.cnki.jjdl.2020.08.013

[72] Shang SZ Yang ZQ. Study on the evaluation of coordinated development of marine economic ecosystem in Jiangsu Province. J View Land Bridge. (2022) 1:31–3.

[73] Cheng N. Research on the evaluation system of sustainable development of China's marine economy under the new normal. J Study Explor. (2017) 5:116–22. 10.3969/j.issn.1002-462X.2017.05.018

[74] Li B Han ZL. Evaluation on the coordination degree of marine economy and socio-economic development in Liaoning Province. J Mar Dev Manag. (2015) 4:93–7. 10.3969/j.issn.1005-9857.2015.04.023

[75] Jiang L. Research on evaluation of comprehensive development strength of regional marine economy in China. J Special Zone Econ. (2018) 3:38–42.

[76] Ding LL Yang Y Li H. Two-way evaluation and difference of high-quality development level of regional marine economy. J Econ Geogr. (2021) 41:31–9. 10.15957/j.cnki.jjdl.2021.07.004

[77] Yu JK Wei Y. Research on vulnerability assessment of marine economic system based on set pair analysis. J Mar Econ. (2016) 6:26–33. 10.3969/j.issn.2095-1647.2016.02.004

[78] Ding LL Zhang Y Xue YM. Research progress on the index system and evaluation method of high-quality development of marine economy. J Mar Econ. (2020) 10:3–16. 10.3969/j.issn.2095-1647.2020.02.001

[79] Wang J Yang K Wang H Chen X. A study on the spatiotemporal coupling between the utilization of marine resources and economic development in China's coastal areas. J Guangdong Ocean Univ. (2016) 36:15–22. 10.3969/j.issn.1673-9159.2016.05.003

[80] Yang XY Yu J. Evaluation of marine ecological environment based on Bayesian Network: A case study of Shandong Province. J Mar Bullet. (2021) 40:473–80. 10.11840/j.issn.1001-6392.2021.04.014

[81] Yu X Hu QG. Evaluation of marine ecological carrying capacity in Zhejiang Province Based on ecosystem health. J Technol Econ. (2017) 30:46–50. 10.14059/j.cnki.cn32-1276n.2017.02.010

[82] Han M Lu G Shi LH Zhang C Yu HZ. Regional comprehensive carrying capacity assessment of Dongying coastal zone. J China Popul Resour Environ. (2017) 27:93–101.

[83] The Central People's Government of the People's Republic of China. Shandong. EB/OL. (2019). p. 1. Available online at: http://www.shandong.gov.cn/art/2022/3/11/art_98093_206404.html

[84] The Central People's Government of the People's Republic of China. Shandong. Entering Shandong. EB/OL. (2022). p. 12. Available online at: http://www.shandong.gov.cn/art/2022/3/11/art_97863_261076.html

[85] General Office of Shandong Provincial People's Government. Notice of the People's Government of Shandong Province on Printing and Distributing the Plan of Shandong Province's Marine Main Functional Area. EB/OL. (2017). p. 9.4. Available online at: http://www.shandong.gov.cn/art/2017/9/4/art_267492_9177.html

[86] Lin XC Liu R. Evaluation of ecological environment carrying capacity of coastal waters in Putian City. J Putian Univ. (2019) 26:94–9.

[87] Liu TW. Carrying Capacity of Resources and Environment in Coastal Waters of Guangdong Province. Guangzhou: Guangdong University of Technology (2021).

[88] Li HQ. On the theory of collaborative governance. J Theory Monthly. (2014) 1:138–42. 10.3969/j.issn.1004-0544.2014.01.032

[89] Ormaza Gonzaìlez FI Castro Rodas D Statham PJ. COVID-19 impacts on beaches and coastal water pollution at selected sites in ecuador, and management proposals post-pandemic. J Front Mar Sci. (2021) 8:669374. 10.3389/fmars.2021.669374

[90] Ding XY He JL. Study on evaluation of marine resources and environmental carrying capacity in Shandong Province. J Mar Econ. (2021) 11:72–9. 10.19426/j.cnki.cn12-1424/p.2021.04.006

[91] Li M Dong A Shao Y Zhang HH Di QB. Evaluation and early warning of sea area carrying capacity in Shandong Province based on multidimensional state space and neural network model. J Mar Bullet. (2015) 34:608–15.

[92] Wang J He HX Chen K Liu Y Zhang Y Ke HW. Comprehensive evaluation of sustainable development capacity of marine ecology in Shandong Peninsula Blue Economic Zone based on DPSIR model. J Mar Sci. (2017) 41:129–36.

[93] Wu GD Gao JG Liu DH. Evaluation of sea area carrying capacity of Shandong Peninsula Blue Economic Zone. J Coastal Eng. (2017) 36:63–70. 10.3969/j.issn.1002-3682.2017.02.008

[94] Wang YM Yu L Chen XX. Assessment of marine ecological carrying capacity in Shandong Province based on SAD-SAS model. J Shandong Indus Commercial Univ. (2022) 36:29–40. 10.3969/j.issn.1672-5956.2022.05.004

